# Labeled As Mentally Ill: Community Perspective Toward Mental Illness in Al Ahsa, Saudi Arabia

**DOI:** 10.7759/cureus.20127

**Published:** 2021-12-03

**Authors:** Saba Firdos, Mohammad Amanullah, Ali O Mobarki

**Affiliations:** 1 Department of Clinical Neuroscience, King Faisal University, College of Medicine, Al Ahsa, SAU; 2 Department of Medicine and Surgery, King Faisal University, Al Hofuf, SAU

**Keywords:** saudi arabia, negative attitude, mental illness, community, beliefs

## Abstract

Background

Communities hold different opinions toward mentally ill people and treat them negatively, irrespective of their behavior. Differences in beliefs can impact not only affected individuals but the entire network and opportunities. This study aimed to examine beliefs about mental illness among different populations of the Saudi community.

Methodology

This descriptive study was conducted among 840 participants from several groups (different family roles, professions, medical and non-medical students, educated and uneducated, etc.) aged 18-75 years in Al Ahsa, Saudi Arabia. Information regarding participants’ demographics and beliefs toward mental illness was collected through validated measures. Data were analyzed using SPSS version 21 (IBM Corp., Armonk, NY, USA).

Results

Data analysis showed that individuals over the age of 30 years believed that mental illness is socially dysfunctional, incurable, and a subject of embarrassment. Married couples also considered mental illness to be socially dysfunctional and a subject of embarrassment. Similarly, unemployed participants had more negative views of mental illness than working individuals and students. Furthermore, educators and other professionals believed that mental illness is more socially dysfunctional than healthcare providers. In addition, community groups with a history of mental illness stated that mental illness is incurable. In the family context, grandparents’ beliefs toward mental illness were more negative than other family members’ views.

Conclusions

This study highlights the diversity in beliefs about mental illness among different groups of Arab society. The influence of poor knowledge, religious beliefs, and subjective attitudes should be explored further, and anti-stigma interventions should be implemented to increase community awareness about mental illness.

## Introduction

Mental illness is a medical condition that includes symptoms affecting an individual’s thoughts, feelings, mood, and behavior [1]. According to the World Health Organization, approximately 25% of the global population is experiencing mental illness [2]. Despite the success of psychiatric treatment, people’s beliefs are still influenced by insufficient knowledge, leading to negative attitudes, self-stigma, and limited help-seeking behavior [3]. Poor mental health literacy is one of the main reasons for stereotyped beliefs, treatment delays, and inadequate use of mental health services [4].

Due to stigma, the label of being mentally ill affects not only patients but also their network; it harms individuals’ work status, marriage proposals, and other opportunities [5]. Cross-cultural studies have revealed the differences in the beliefs towards mental illness, such as Asians prefer to deal with emotional problems through biomedical treatment [6]. However, Southeast Asians perceive that supernatural forces are accountable for mental health issues and consider them the result of wrath and denial of spirit or deities [7], reporting negative perception toward mental illness [8].

Stigma toward people with mental illness exists among Arabs in the Middle East [9] with considerable diversity [10]. Existing studies of mental illness in Saudi Arabia have estimated that 16.3% of Saudi females suffer from psychological symptoms [11]. However, the prevalence of mental disorders was estimated to be 48% in a similar population [12]. In particular, women held more negative beliefs toward mental illness in Arab society [13].

Perception and attitudes towards mental illness in Arab communities are generally influenced by pre-existing beliefs based on their experiences [14]. Hence, it is important to address mental illness beliefs, perceptions, and risk factors among such communities with limited resources [15]. Thus, this study aims to determine the beliefs toward mental illness among different populations of Saudi communities.

## Materials and methods

Sample and procedure

A community-based, cross-sectional study was conducted among 840 participants from different categories (age, family roles, professions, medical and non-medical students, history of mental illness, educated and uneducated, etc.) aged 18-75 years in the Al Ahsa governorate in the eastern province of Saudi Arabia. For sampling, with the assumption of an error of 5%, 95% confidence interval, and 50% proportion was used to estimate the minimum sample size for the study. The participants were invited and sampled electronically. They were requested to fill an online questionnaire via Google Forms as it was found to be a valid approach for data collection [16]. The primary ethical issues in this study were informed consent, confidentiality, withdrawal rights, anonymity, privacy protection, and maintaining the dignity of all participants. An institutional review committee of Research Ethics, College of Medicine, King Faisal University, Al Ahsa, Kingdom of Saudi Arabia, approved the study (2020-10-34).

Measures

Sociodemographic Information

A background sheet was prepared to obtain personal information (age, gender, marital status, education, family role and status, type of job, and history of mental illness).

Beliefs Toward Mental Illness Scale

The Beliefs Toward Mental Illness (BMI) scale [17] consists of 21 items that measure the negative stereotypical views of mental illness as dangerous, socially dysfunctional, incurable, and embarrassing. The scale was rated on a six-point Likert-type scale ranging from zero (strongly disagree) to five (strongly agree). The BMI reported good alpha reliability coefficients for four-factor ranging from 0.70 to 0.84.

Statistical analysis

Categorical variables are presented as counts and proportions (%), whereas continuous variables are presented as a minimum, maximum, mean, and standard deviation, whenever appropriate. Between comparison of variables, Mann-Whitney U test or Kruskal Wallis test was applied. Statistical collinearity (i.e., inflation factor) was assessed using Kolmogorov-Smirnov and Shapiro-Wilk tests. Because all data comparisons followed the abnormal distribution, non-parametric tests were applied. A p-value of ≤0.05 (two-sided) was used to indicate statistical significance. All data analyses were performed using the Statistical Packages for Software Sciences (SPSS) version 21 (IBM Corp., Armonk, NY, USA).

## Results

Table 1 presents the sociodemographic characteristics of participants. More than half (55.6%) of the participants were in the younger age group, and approximately 55% were females. Concerning marital status, 51.7% of the participants were not married. Over two-thirds (69.6%) of the participants were professionals. Furthermore, the most frequently mentioned role in the family was a sister (25.6%), followed by a brother (25.2%) and a mother (24.4%). Concerning their employment status, 36.1% were officers, while 42.4% were students. Of the employed participants, 40.8% were teachers, and 32.4% were administrative employees. The prevalence of a family history of mental illness was 34.4%.

**Table 1:** Sociodemographic characteristics of participants (N = 840).

Study variables	n	%
Age group		
≤30 years	467	55.6
>30 years	373	44.4
Gender		
Male	381	45.4
Female	459	54.6
Marital status		
Unmarried	434	51.7
Married	406	48.3
Role in the family		
Husband	09	1.1
Wife	32	3.8
Father	158	18.8
Mother	205	24.4
Sister	215	25.6
Brother	212	25.2
Grandparent	09	1.1
Educational level		
Diploma or below	255	30.4
Bachelor or higher	585	69.6
Employment status		
Officer	303	36.1
Housewife	86	10.2
Student	356	42.4
Unemployed	65	7.7
Free business	30	3.6
Type of job (n = 333)		
Teacher	136	40.8
Administrative employee	108	32.4
Healthcare provider	11	3.3
Other	78	23.4
Family history of mental illness		
Yes	299	34.4
No	551	65.6

When measuring the differences between BMI and its subscales among the sociodemographic characteristics of participants, Table 2 shows that the mean score of the older age group (>30 years) was significantly higher among subscales of social dysfunction (p < 0.001), incurability (p < 0.001), embarrassment (p < 0.001), and total BMI (p < 0.001). The beliefs of married respondents showed significantly higher social dysfunction (p < 0.001), embarrassment (p < 0.001), and total BMI (p < 0.001). Participants with a family history of mental illness were observed to have statistically significantly higher incurability beliefs (p < 0.001) toward mental illness. On the other hand, the beliefs of students were significantly low regarding dangerousness (p < 0.05), social dysfunction (p < 0.001), incurability (p < 0.05), embarrassment (p < 0.001), and BMI (p < 0.001). The mean score of healthcare providers on the subscale was also significantly low regarding social dysfunction (p < 0.05) and total mean BMI scores (p < 0.05) than other professions.

**Table 2:** Comparison of BMI and its subscales with the sociodemographic characteristics of participants (N = 840). ^a^: P-value has been calculated using the Mann-Whitney U test. ^b^: P-value has been calculated using the Kruskal-Wallis test. **: Significant at p ≤ 0.05. BMI: Beliefs Towards Mental Illness; SD: standard deviation

Characteristics	Dangerousness		Social dysfunction		Incurability		Embarrassment		Total BMI scale score	
	Mean	SD	Mean	SD	Mean	SD	Mean	SD	Mean	SD
Age group^a^										
≤30 years	9.09	4.07	17.5	6.13	13.9	4.57	6.86	4.51	47.4	14.9
>30 years	9.67	3.82	20.5	6.56	15.1	4.51	8.09	4.18	53.3	15.2
t-test	-2.092		-6.830		-3.615		-4.062		-5.699	
P-value	0.051		<0.001^**^		<0.001^**^		<0.001^**^		<0.001^**^	
Gender^a^										
Male	9.23	3.74	18.8	6.52	14.3	4.42	7.74	4.48	50.1	15.2
Female	9.45	4.16	18.9	6.48	14.5	4.71	7.13	4.34	50.0	15.4
t-test	-0.808		-0.254		-0.706		2.005		0.047	
P-value	0.425		0.834		0.698		0.066		0.915	
Marital statuse^a^										
Unmarried	9.07	4.08	17.9	6.51	14.2	4.70	6.85	4.50	48.1	15.4
Married	9.65	3.84	19.8	6.35	14.6	4.44	7.99	4.24	52.1	15.0
t-test	-2.123		-4.150		0.105		-3.791		-3.825	
P-value	0.054		<0.001^**^		0.052		<0.001^**^		<0.001^**^	
Educational level^a^										
Diploma or below	9.54	4.08	19.1	6.49	14.5	4.50	7.09	4.34	50.3	15.2
Bachelor or higher	9.27	3.93	18.8	6.49	14.4	4.62	7.54	4.44	49.9	15.4
t-test	0.902		0.727		0.412		-1.354		0.276	
P-value	0.539		0.567		0.284		0.186		0.799	
Current employment status^b^										
Employed	9.26	3.79	19.8	6.88	14.8	4.83	7.97	4.34	51.8	16.1
Unemployed	10.0	3.72	19.9	6.11	14.9	4.29	7.84	4.32	52.6	13.9
Student	9.16	4.21	17.6	6.05	13.9	4.41	6.69	4.42	47.3	14.6
F-test	2.628		12.652		4.243		8.309		10.240	
P-value	0.050^**^		<0.001^**^		0.006^**^		<0.001^**^		<0.001^**^	
Type of job (n = 333)^b^										
Teacher	9.50	3.90	20.5	6.67	15.2	5.05	7.85	4.05	52.7	15.7
Administrative employee	9.05	4.03	19.3	6.89	13.9	4.99	7.83	4.34	50.3	17.1
Healthcare provider	6.91	3.42	13.7	6.71	13.3	5.33	5.73	5.29	37.3	16.2
Other	9.45	3.19	20.1	6.93	14.8	4.13	8.04	4.55	53.1	14.9
F-test	1.781		3.555		1.618		0.937		3.638	
P-value	0.212		0.026^**^		0.211		0.454		0.021^**^	
Student (n = 356)^a^										
Medical	8.67	4.04	17.3	6.41	13.9	4.42	6.71	4.37	46.7	15.1
Non-medical	9.56	4.33	17.8	5.75	13.8	4.41	6.68	4.48	47.8	14.3
t-test	-1.990		-0.631		0.366		0.073		-0.698	
P-value	0.061		0.642		0.526		0.984		0.430	
Family history of mental illness^a^										
Yes	9.00	4.26	18.8	6.69	15.3	4.67	7.25	4.43	50.3	15.2
No	9.53	3.80	18.9	6.39	13.9	4.46	7.49	4.39	49.9	15.4
t-test	-1.852		-0.228		4.080		-0.746		0.417	
P-value	0.076		0.739		<0.001^**^		0.446		0.842	

On comparing BMI and its subscales with respect to family roles, being a sister was significantly low on the subscale of embarrassment (p < 0.001), and total BMI (p < 0.05) was also lower than other relations. However, the mean score for embarrassment among grandparents was significantly higher, along with overall BMI scores (Table 3).

**Table 3:** Comparison of BMI and its subscales with the participant’s role in the family (N = 840). P-value has been calculated using the Kruskal-Wallis test. **: Significant at p < 0.05. BMI: Beliefs Towards Mental Illness; SD: standard deviation

Role in the family	Factor 1		Factor 2		Factor 3		Factor 4		Total BMI scale score	
	Mean	SD	Mean	SD	Mean	SD	Mean	SD	Mean	SD
Husband	9.00	4.95	19.4	7.57	14.0	4.09	8.11	4.76	50.6	16.2
Wife	9.78	2.94	18.3	4.93	14.2	3.79	7.31	3.88	49.6	10.9
Father	9.43	3.54	19.2	6.73	14.3	4.87	8.14	4.31	51.0	15.7
Mother	9.96	3.99	19.9	6.51	14.7	4.74	8.17	4.12	52.8	15.5
Grandparents	9.78	4.21	21.0	5.59	16.4	3.84	8.89	4.68	56.1	16.2
Sister	8.92	4.43	18.1	6.34	14.3	4.74	6.10	4.36	47.4	15.2
Brother	9.08	3.81	18.4	6.56	14.3	4.19	7.37	4.59	49.2	15.0
F-test	1.499		1.866		0.539		5.242		2.669	
P-value	0.175		0.125		0.802		<0.001^**^		0.019^**^	

## Discussion

The goal of this study was to investigate the beliefs of the Saudi community toward mental illness. Because up to 70% of people with a mental illness do not seek medical treatment [18] due to stigmatization, cultural beliefs, and poor knowledge, these factors are more likely to influence an individual’s perception, attitude, and experience. Other influences, such as religious beliefs, misconceptions, and social beliefs, are also significant factors behind the stereotypical opinion toward people with a mental health condition irrespective of their behavior. This scenario has also been observed in Saudi Arabia, where mental illness is associated with strong beliefs in supernatural powers (e.g., the evil eye and Jinn) [19].

Interestingly, several studies have reported the significant progress of psychiatry services in Saudi Arabia over the past decades concerning specialized hospitals, health services, and specialists [20]. Nevertheless, insufficient knowledge can cause negative attitudes and limit help-seeking behavior, leading to self-stigmatization [4]. Our findings (Table 2) showed that the population above the age of 30 has overall negative beliefs toward mental illness. They consider mental illness to be more socially dysfunctional, incurable, and a matter of embarrassment. A possible reason for this finding may be insufficient knowledge about mental illness, which leads to misconceptions as the Saudi population over the age of 18 previously reported negative attitudes toward help-seeking behaviors for mental illnesses [21]. Similar to this finding in a large hospital, most participants reported negative views about mentally ill patients as dangerous and reported discriminative behavior [22].

Similarly, married participants also believed mental illness to be a cause of social dysfunction and embarrassment (Table 2). Stigmatized labeling of mental illness as a factor of fear, avoidance, and hostile behavior can lead to unemployment and social isolation among individuals with a mental health condition [23].

The current status significantly influences community beliefs as our findings showed that unemployed participants had more negative views toward mental illness than employed participants and students (Table 2). They believed that mental illness is dangerous, socially dysfunctional, and incurable, except in the subscale of embarrassment. Similar to our findings, a community-based study reported mental illness as superstitious and a reason for unemployment [24]. Employed participants felt embarrassed with mental illness rather than as being dangerous, socially dysfunctional, and incurable. Students were observed to have significantly fewer stereotypical beliefs about mental illness and its subscales. Consistent with our findings, variations were observed in various studies as negative opinions were seen in young adults despite having a positive attitude toward help-seeking behaviors in mental illnesses [25]. Unlike our finding, a previous study showed that undergraduate students had positive attitudes related to mental illness [26]. Nevertheless, some growing evidence shows a mixed opinion of students toward mental illness [27].

Surprisingly, teachers and other professions had shared conservative opinions for mental illness than healthcare providers and administrative employees. Their beliefs on mental illness were seen as more socially dysfunctional in comparison to other subscales; however, overall mental illness beliefs of different professions were more stereotypical. The most plausible reason may be the background of healthcare workers. Previous studies have shown that employment and gender are positively correlated with help-seeking behaviors for mental health conditions [21].

Presumably, mental illness leads to feelings of shame and burden in affected families, leading to rejection and exacerbation of their social status [28]. Study participants with a family history of illness reported statistically significant incurable beliefs. Similar to our findings, hesitation, negative attitudes, and less cost-effective estimations regarding mental illness treatment have been reported in Saudi Arabia [21].

Furthermore, as shown in Table 3, family members were significantly different in terms of their beliefs on the subscales of embarrassment. Grandparents considered mental illness as more embarrassing compared to other family members. It is difficult to relate this finding with previous studies due to limited data. Some studies have reported that people consider patients with mental illnesses as dangerous, unsuitable for friendship [19,21], and maintain a hesitant attitude [29]. Negative beliefs about mental illness in Saudi Arabia are due to poor or insufficient mental health literacy [27]. However, contradictory evidence shows that many Saudi adults believe that patients with mental illnesses deserve respect and proper treatment [30]. Additionally, there is growing, but limited, evidence regarding the changes in the attitudes and perception toward the stigma and treatment of mental illnesses in Saudi Arabia.

Limitations

This study is one of the few studies conducted to explore the views of mental illnesses among different categories of the Saudi community. However, some factors can generate the possibility of errors. For wide-ranging results, the approach should reach the maximum number of responses to explore the existing scenario. Because this study was conducted through an online platform and was limited to one city, the scope of the representativeness of the findings is narrow. Other towns, different populations, various measures, and scenarios may increase the generalizability of the findings to represent the knowledge or beliefs toward mental illness.

## Conclusions

The findings of this study contribute significantly to demonstrating the community’s beliefs toward mental illness. It recognizes that various groups of the community have different opinions. Undoubtedly, this can be associated with the increased stigma and discrimination toward people with mental health conditions. Public awareness and further investigation are needed to ascertain the factors and gaps responsible for negative opinions related to mental illness. Better knowledge, positive attitudes, and appropriate use of the services can improve the community’s perception. Thus, we recommend that future studies should focus on qualitative and quantitative approaches to represent a broader sample of the community concerning the perception of help-seeking behavior and knowledge of mental illnesses.
